# Crystallisation Behaviour of Pharmaceutical Compounds Confined within Mesoporous Silicon

**DOI:** 10.3390/pharmaceutics12030214

**Published:** 2020-03-02

**Authors:** Eleanor C. L. Jones, Luis M. Bimbo

**Affiliations:** Strathclyde Institute of Pharmacy and Biomedical Sciences, University of Strathclyde, 161 Cathedral Street, Glasgow G4 0RE, UK; eleanor.jones@strath.ac.uk

**Keywords:** crystallisation, porous silicon, drug confinement, drug delivery

## Abstract

The poor aqueous solubility of new and existing drug compounds represents a significant challenge in pharmaceutical development, with numerous strategies currently being pursued to address this issue. Amorphous solids lack the repeating array of atoms in the structure and present greater free energy than their crystalline counterparts, which in turn enhances the solubility of the compound. The loading of drug compounds into porous materials has been described as a promising approach for the stabilisation of the amorphous state but is dependent on many factors, including pore size and surface chemistry of the substrate material. This review looks at the applications of mesoporous materials in the confinement of pharmaceutical compounds to increase their dissolution rate or modify their release and the influence of varying pore size to crystallise metastable polymorphs. We focus our attention on mesoporous silicon, due to the ability of its surface to be easily modified, enabling it to be stabilised and functionalised for the loading of various drug compounds. The use of neutron and synchrotron X-ray to examine compounds and the mesoporous materials in which they are confined is also discussed, moving away from the conventional analysis methods.

## 1. Introduction

### 1.1. Drug Crystallisation and Amorphisation 

Nearly half of all the newly developed compounds entering the drug development pipeline suffer from unfavourable physicochemical properties which affect their bioavailability and, consequently, the efficacy of the treatment [1]. Poor aqueous solubility is one of the most prevalent issues affecting both the drug’s pharmaceutical processability and biodistribution and is intimately tied to the compound’s solid-state properties. The solid phase can be subcategorised into two divisions according to their order of molecular packing/arrangement: the crystalline and amorphous states. 

**Figure 1:**
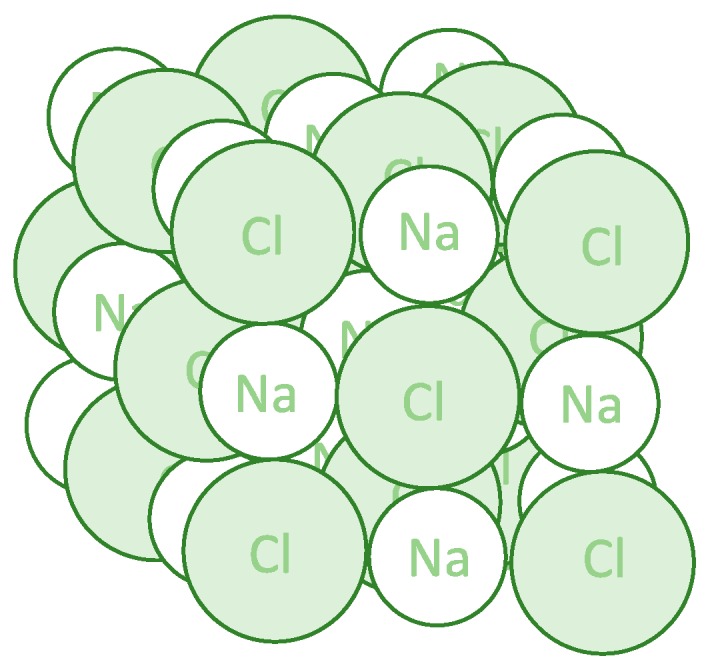
Three-dimensional structure of the sodium chloride crystal. Each sodium ion is octahedrally surrounded by six chloride ions, and each chloride ion is octahedrally surrounded by six sodium ions.

Crystalline materials are ordered in a repeating pattern, creating a three-dimensional crystal structure held together by intermolecular forces, including hydrogen bonding and van der Waals forces. The smallest repeating unit is known as the unit cell and, when built up, shows the composition of the crystal (Figure 1). Crystalline materials are at their most stable state with atoms packed in a way that reduces their total potential energy [2]. Due to their high stability, a large amount of energy is needed to break the intermolecular forces in the crystal structure. When crystalline solids are melted, they produce a well-defined melting point, determining the energy needed to break down the crystal structure. 

Crystallisation is a process that allows for the formation and purification of many active pharmaceutical ingredients (APIs), generating solid particles with the desired morphology, crystal form and purity, crucial for controlling a compound’s physicochemical properties. The process can be divided into two subprocesses: nucleation and crystal growth. Nucleation is defined as the formation of small “crystalline entities”, nuclei, from a supersaturated solution [3,4]. The critical nucleus size is said to fall within the range of 100 to 1000 atoms, and once reached, crystal growth can follow with the growth of nuclei into “macroscopic crystals” [5]. Nucleation can be classified as primary, occurring in the absence of crystalline material, or secondary, which occurs in its presence and often serves as a catalyst for further nucleation. Primary nucleation can be classified as either homogeneous or heterogeneous. Heterogeneous nucleation occurs at the interface of a surface, whereas homogeneous nucleation occurs in a clear solution [6]. 

The crystalline state can be composed of multiple components, e.g., co-crystals, solvates and hydrates, or a single component, polymorphs. Polymorphism is the ability of a compound to exist in more than one crystalline form, with molecules arranging themselves differently within the crystal structure, attributing to different physical and mechanical properties between forms. Polymorphs of the same material can exhibit different melting points, crystal habits and solubilities, which is of great interest and concern to the pharmaceutical industry. Solubility differences between polymorphs can be explained by the difference in lattice energy, i.e., the attractive forces that hold the crystal structure together and repulsive forces that allow the crystal to dissociate in solution. It is understood that metastable polymorphs display greater solubility but can undergo solvent-mediated transformation to a more stable, hence lower, solubility state in order to retain system equilibrium [7]. Unfortunately, due to the nature of metastable polymorphs, pharmaceutical formulations often contain the most stable form. Understanding the factors that play a role in polymorphism is important, with screening of crystalline materials playing a fundamental role in drug development. During solution crystallisation, the metastable polymorph should crystallise first, according to Ostwald’s rule of stages, with subsequent transformation to the stable form [8].

Conversely, amorphous materials lack the long-range order that crystalline materials possess. The irregular arrangement of molecules leads to unequal intermolecular forces and, hence, physical instability in comparison to their crystalline counterparts. Amorphous compounds possess a greater Gibbs free energy than their crystalline state and are thermodynamically unfavourable, which can lead to their transformation to the stable crystalline state [9]. This instability is advantageous in terms of increased solubility of compounds and, hence, bioavailability. A higher energy state often increases the mobility of the molecules, giving the compound a greater solubility. However, it is difficult to determine the solubility of amorphous compounds due to their tendency to recrystallise upon contact with an aqueous environment [10].

### 1.2. Drug Confinement into Porous Substrates

As mentioned, amorphous compounds display greater solubility than their stable crystalline counterparts due to higher free energy and greater molecular mobility. Nonetheless, their tendency to recrystallise can eliminate this highly desirable property [11]. The vast majority of new and existing drug compounds are crystalline, with their long-range order promoting stability. When confined in pores, however, they tend to lose their long-range order, remaining in a disordered state, thus reducing their thermodynamic stability and improving dissolution from the substrate in aqueous environments [12,13]. Inorganic mesoporous substrates, i.e., materials with pores ranging between 2 and 50 nm in diameter, have shown promise for stabilising drugs in the amorphous state due to their exceptional properties, such as a large pore volume, which confers the ability to host large amounts of drug compounds; a high surface area, allowing great potential for drug adsorption; and tuneable pore size with an uniform distribution, allowing a reproducible loading and release of drugs [14,15,16,17]. The amorphisation of pore-loaded compounds is hypothesised to be related, amongst other properties, with the limited space available within a pore, which can prevent nucleation and subsequent crystal growth by decreasing Gibbs free energy, leading to the confinement and stabilisation of the drug [18,19,20]. The confinement of drug compounds inside the pores of mesoporous materials additionally enables the attainment of local high-drug concentrations and even drug supersaturation, leading to an increase in drug permeation and subsequent net absorption [21,22]. This has allowed drug confinement into mesoporous materials to emerge as an attractive strategy to improve the dissolution behaviour of poorly water-soluble compounds. Thus, understanding the crystallisation (or lack of) behaviour of drug compounds confined within these substrates at a molecular level is of critical importance in the formulation of amorphous systems [23,24].

The polymorphism of crystalline compounds has been investigated with the aid of porous substrates with a view to manipulating the nucleation process [25,26]. As mentioned, crystallisation in pores can be regulated by changing the pore size to dimensions close to that of the critical nucleus size, therefore regulating polymorphism if each form has a different critical nucleus size. It has also been stated that pore sizes should be approximately 20 times the molecular radius of the compound for crystallisation to occur in confinement [27]. Dwyer and collaborators referred to this finding when discussing the results of fenofibrate confined in nanoporous silica. Amorphous fenofibrate was discovered in pores less than 20 nm, but crystalline in pore sizes greater than 20 nm [28]. According to the molecular radius of fenofibrate (~1.27 nm), the drug would not have enough space in the 20 nm pore to crystallise and would therefore be amorphous. The large surface area of the porous substrate could also regulate crystallisation by providing a surface for heterogeneous nucleation on the pore walls.

**Figure 2:**
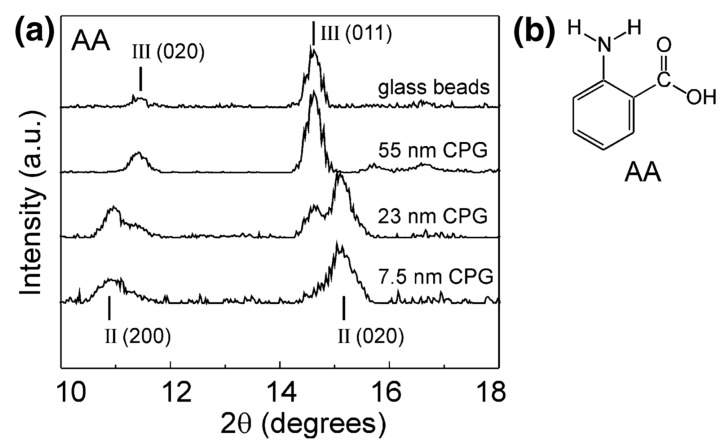
(**a**) X-ray powder diffraction (XRPD) data for anthranilic acid crystallised by cooling of its melt on nonporous glass beads and within controlled pore glasses (CPGs) of various pore sizes. (**b**) Structure of anthranilic acid (AA). Reproduced with permission from [25].

The polymorphism of anthranilic acid (AA), for example, known to exhibit three polymorphs, was investigated using porous and nonporous glass as crystallisation templates. Form I was mixed with nonporous glass beads and controlled pore glass (CPG) and heated above the melting point of the acid. Form III, the metastable polymorph known to crystallise from the melt, was discovered on the surface of the nonporous glass and confined within a controlled pore glass of 55 nm. Form II was dominant in pores of 7.5 nm, with the nanoscale crystals depicted by the decrease in melting point of anthranilic acid and broadening of the X-ray diffraction (XRD) peaks (Figure 2). Confinement led to the stabilisation of the metastable polymorph, with storage studies conducted for one month at room temperature. The authors suggested that the confinement of metastable Form II was due to the critical nucleus size being suitable for the smaller pore sizes but could also have been due to the influence of pore size on heterogeneous nucleation [25].

Among pharmaceutically relevant compounds, indomethacin has been extensively employed as a model drug when investigating the influence of confinement on drug molecules, due to its array of polymorphs and limited aqueous solubility. Nartowski and collaborators investigated the influence of pore size and drug-loading method on the polymorphism of indomethacin. The CPG and mesoscopic cellular foam (MCF) were used as the substrates due to their varying pore sizes. Loading from the melt led to the identification of amorphous indomethacin within the porous materials, displaying an amorphous “halo” in the X-ray powder diffraction (XRPD) patterns. Solid-state nuclear magnetic resonance (NMR) was also utilised, with the broadening of indomethacin peaks in the spectra indicating the many orientations of carbon atoms and differences in environments within the amorphous sample. The smaller pore size of MCF prevented the thermally induced recrystallisation of indomethacin; however, the larger pore size of CPG allowed thermally induced recrystallisation at higher concentrations. This highlights the importance of drug concentration when loading the porous material, as well as pore dimensions [29]. The addition of solvent to the melt-loaded MCF caused molecular rearrangement in indomethacin, driving the drug’s transition from amorphous to crystalline. Increased drug-loading was observed with the increase in total pore volume and surface area. After the addition of methanol to the loaded CPG, the crystallisation of multiple polymorphs was identified, with the addition recrystallising pure form V from the amorphous state, usually produced from the desolvation of the methanol-indomethacin solvate.

The influence of pore volume and surface area on drug-loading capacity was also investigated by Bavnhøj and collaborators using three model drugs with good glass-forming capabilities [18]. Loading capacity was defined to be either monomolecular loading capacity (MLC) or pore-filling capacity (PFC), and differential scanning calorimetry (DSC) was used to determine the MLC defining the heat capacity over the glass transition temperature from the amorphous state of each drug. Pore volume was determined to be the limiting factor to drug-loading capacity in this case. Increase in surface area from 550 m^2^/g to 681 m^2^/g did not increase the experimental MLC value, as greater surface area was produced at the expense of pore size, leading to spatial limitation. The theoretical MLC suggested the general trend to be an increase in MLC with an increase in surface area, which assumed the entire silica surface to be covered by drug molecules and did not account for the blocking of pores due the molecular size of the drugs. By plotting the experimental MLC along with the theoretical MLC and PFC as a function of surface area and pore volume, the authors were able to identify their influence on drug-loading fraction, with four zones describing loading within the pores or on the surface of the mesoporous silica, which could prove beneficial to future investigations looking into compound-loading onto mesoporous materials.

### 1.3. Crystallisation Characterisation Techniques

Thermal evaluation of the effect of confinement on drug compounds can be provided by DSC, whilst also offering fast benchtop analysis. This technique can be used to quantify and differentiate between drug molecules confined in porous materials and that on the surface of the material [30]. It can also differentiate between crystalline and amorphous material. Kiwilsza and collaborators used DSC to determine the surface crystallisation of nimodipine in porous silica [17]. The endotherm indicated the melting of crystalline nimodipine on the surface of the porous silica when unwashed. Washing the loaded silica removed the surface crystalline material, which was indicated by the lack of endotherm. As no melting peak was observed for the washed samples, it was inferred that the confined nimodipine was amorphous. To confirm that the washed particles were loaded with nimodipine, the authors carried out a drug dissolution study. More than 60% of the drug was reported to dissolve and be released within the first three minutes of the study. Dwyer and collaborators took a similar approach, using DSC in the analysis of confined fenofibrate [28]. The sharp single melting endotherm indicated crystalline fenofibrate, but the lack of melting point for the smallest pore size indicated amorphous fenofibrate, which was confirmed by NMR. Double peaks in the DSC thermograph would speculate surface crystallisation in addition to confined crystals. 

The XRPD offers a complementary way of assessing the crystallinity of confined compounds [19,25,28,29,31,32,33]. Like DSC, XRPD can be used to determine if the confined drug is in its crystalline or amorphous state. If the drug is crystalline, diffraction peaks will be observed in the pattern, and if well-defined, they can be used to determine the crystal structure present using crystallographic software. It can also illustrate if a mixture of crystal systems are present when dealing with polymorphs [31]. One of the disadvantages of XRPD is the decrease in peak intensity at greater angles. A way to overcome this is through neutron powder diffraction, which relies on the same principles as XRPD. Whilst X-rays interact with the electrons in the sample, neutrons interact with the nuclei. This means that neutron diffraction is better at resolving the hydrogen positions of a drug compound, for example, or can be used to investigate surface interactions between the drug and the pores it is confined within. Unfortunately, powder neutron diffraction requires the drug compounds to be deuterated, where the hydrogen atoms are replaced with the isotope deuterium, which may not always be technically feasible. Analysis by neutron can only be carried out in specialised research facilities: for example, ISIS Neutron and Muon Source in the UK and the Institut Laue-Langevin located in France. 

Aside from DSC and XRPD methods, neutrons and synchrotron X-rays have shown promise as techniques used to investigate porous substrates. Webber and Dore used neutron diffraction cryoporometry to assess the crystallisation behaviour of water and ice in porous silica. They were able to differentiate between the different states, observing that the signal produced was proportional to the quantity of the liquid or solid crystalline state, hence, were able to track the ratio as a function of temperature. From this, the Gibbs-Thomson equation could be applied to interpret structural information of the compound’s state when confined in SBA-15, a type of mesoporous silica nanoparticle [34]. 

The confinement of proteins in mesoporous silica was also investigated using small-angle neutron scattering (SANS), assessing their arrangement within the pores. Again SBA-15 was chosen for its well-defined geometrical pores, and KIT-6 mesoporous silica was chosen for the contorted nature of the pores, allowing for the comparison of protein arrangement with regards to pore morphology [35]. The authors first charged the proteins to allow for electrostatic attraction with the silica substrates. They then observed that lower protein concentrations showed lower signal amplitudes from additional protein adsorbed on the walls of the silica matrix or free inside the pores, which distorted the scattering and, hence, substantiated that the information produced from SANS was reliable. Further research detailed the use of deep inelastic neutron scattering (DINS) to investigate mesoporous silica, due to its sensitivity for the investigation of the local environment of protons, complementing diffraction studies on atomic spatial distributions [36]. Water molecules were confined within hexagonally arranged porous silica with pore sizes of 4.3 nm. This led to the conclusion that hydrogen bonding between the water molecules and the silanol groups was much stronger than the hydrogen bonds within unconfined water, supporting the hypothesis of a difference in proton environment. 

Pair distribution function (PDF) has also been used to probe the local atomic ordering of SBA-15 porous silica by investigating the relationship between porous structure and thermal stability of the substrate. Atomic pair distribution is the sine Fourier transform of the structure function that is determined experimentally by X-ray or neutron diffraction and can be used to study materials at an atomic scale [37]. Rantanen and collaborators used small-angle X-ray diffraction (SAX) to determine the distance between the pores and, hence, the pore wall thickness. The SAX diffraction data further showed that, with decreasing pore size, the position of the (100) peak of silica shifted to a lower 2θ. Decrease in pore size also showed an increase in wall thickness and a lower order of structure possessed by the walls. The measured structure function showed differences between pore sizes, with greater separation between peaks for the samples with larger pore sizes (9.69 and 12.05 nm average diameter), suggesting that the ordering of SiO_4_ tetrahedra was more regular. There was also an indication that silicon atom pairs were separated by a lesser distance, causing the structure to be more tightly packed, resulting in a greater density at the pore walls, which could influence the stability of the porous structure as a whole [37]. The PDF analysis has also been used to investigate the changes in short- to medium-range structures of nanoporous silica, characterising pore morphology as well as pore wall thickness and atomic structure within the pore walls [37,38].

Detailed structural information can also be gained from using X-ray total scattering coupled to atomic PDF and solid-state NMR. Atomic PDF is well-suited for structural characterisation of crystalline materials, as well as nano-sized and amorphous materials. It has been stated that PDF is a representation of “total scattered X-ray intensities and reflects both long-range atomic structure, manifested in the sharp Bragg peaks, and the local structural imperfections, manifested in the diffuse components of the total scattering pattern” [39]. The structural information that PDF provides persists over long distances and can therefore describe structural properties at varying orders of scale. However, structural studies based on PDFs obtained from total X-ray scattering data of amorphous organic-inorganic nanocomposites can prove challenging due to their weak scattering power.

**Figure 3:**
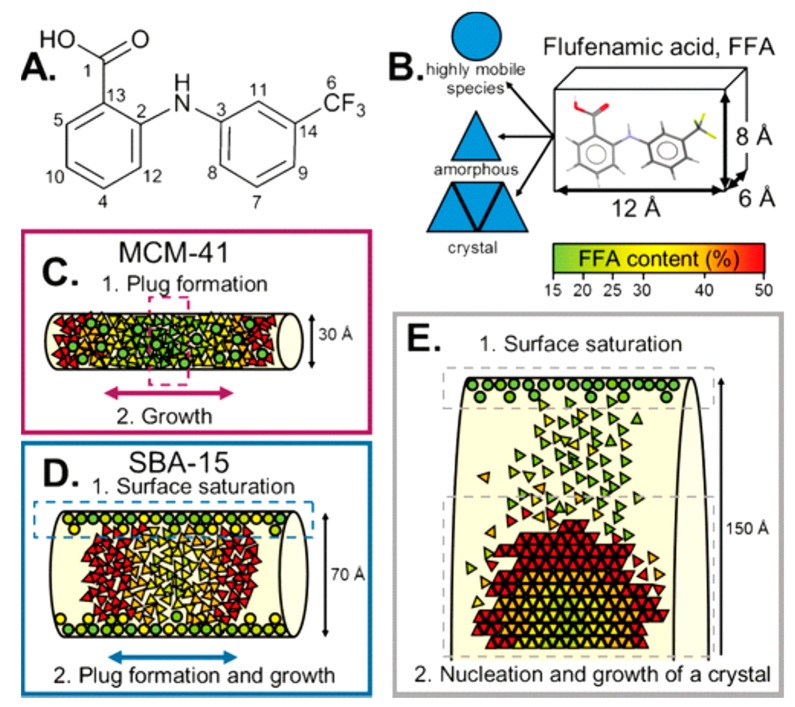
(**A**) Structure of flufenamic acid (FFA) with labelling of the carbon atoms. (**B**) Average dimensions of the FFA molecule and the three different states of FFA species inside the pores. (**C**,**D**) Different mechanisms for FFA adsorption and stabilisation of the amorphous state. (**E**) Mechanism of the formation of crystalline FFA form I in mesoscopic cellular foam (MCF). Reproduced with permission from [40].

An application of such principles was explored by Hsieh and collaborators, where they investigated mononitrosyl complexes in porous silica matrices to determine a structural characterisation strategy to apply to another compound, sodium nitroprusside. As with any investigation, complementary methods of analysis are needed, and in this study, short-range NMR and long-range XRD provided the orthogonal approach. Thus, PDF can be used to bridge the gap between the two methods. The XRD data of loaded and unloaded silica showed amorphous features within the diffraction patterns with a characteristic peak at Q ~1.64 Å. Change in scattering contrast was witnessed between unloaded and loaded samples, with the principal peak within the loaded diffraction pattern displaying a broader nature with a lower intensity [39]. In a study by Nartowski and collaborators, ^19^F solid-state NMR together with terahertz spectroscopy was used to detect the presence of confined molecules of flufenamic acid (FFA) in different environments and motional regimes [40]. This enabled the authors to gain in situ mechanistic insight into the molecular self-assembly at different length scales by taking advantage of the higher sensitivity of ^19^F nuclei to changes in the local environments of molecules confined in the porous hosts. The authors reported for the first time the presence of a liquid-like layer of drug molecules on the porous scaffold’s surface, which affected the nucleation and crystallisation behaviour of the drug (Figure 3).

NMR has also been utilised to characterise the porous structure of hydrogels. Average pore diameter and pore volume are usually determined using mercury porosimetry and gas adsorption, but due to the nature of the hydrogel, NMR provides an alternative that is not impeded by the liquid phase. Low-field NMR—pulsed gradient spin echo was employed to understand the pore characteristics of a hydrogel composed of bacterial cellulose and acrylic acid, with high diffusion times indicating the interconnected nature of hydrogel pores [41]. NMR relaxation times have also been used to determine pore radius distribution profiles of hydrogels quickly without destroying the sample. NMR measurements rely on the excitation of targeted nuclei spins in the aqueous phase and subsequent measurement of the proton spins returning to equilibrium [42]. The relaxation rate of proton spins near the pore’s surface is faster than in the bulk, providing a difference which can be measured. Sørland and collaborators used NMR to investigate pore size distribution of porous rocks, further concluding that NMR was more sensitive to pore size distributions than the mercury intrusion technique, as mercury underestimates larger cavities [43]. 

### 1.4. Porous Silicon 

One of the materials that has shown great promise for the confinement of drugs is porous silicon (pSi). When pores are introduced into the structure of the chemical element silicon, rendering a large surface-to-volume ratio, we obtain the pSi form. The discovery of pSi was made fortuitously in 1956 while trying to develop a method for shaping and polishing the surfaces of silicon and germanium [44]. At the time, their porous nature was not reported, and only several years later, in 1971, a procedure conducive to obtaining highly microporous silicon was published [45]. However, it was not until the early 1990s when Leigh Canham, concurrently with Lehmann and Göselle, hypothesised that the thin silicon filaments, created when the pores become large and numerous enough to overlap, might display quantum confinement effects, leading to the demonstration that silicon wafers could emit light if subjected to chemical and electrochemical dissolution [46,47]. These discoveries soon instigated a substantial amount of research focused on Si-based lasers, displays, and optoelectronic switches. Nonetheless, due to the material’s mechanical and chemical instability, as well as its low electroluminescence efficiency, most of the research in that area subsequently faded. In addition to its electronic properties, pSi was later found to act as a bioactive material [48], which rekindled its study for biomedical applications [49,50,51,52,53,54]. 

**Figure 4:**
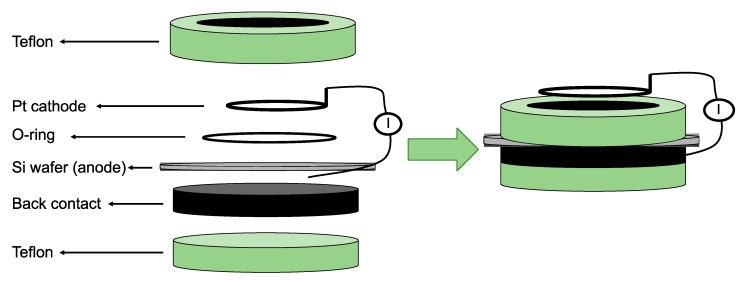
Schematic representation of a one-side etching setup for porous silicon (pSi) fabrication. The Pt is the cathode and the Si wafer the anode in a hydrofluoric acid (HF) ethanolic solution. Reproduced with permission from [57].

There are over 40 different fabrication routes for pSi, using both “top-down” and bottom-up” approaches. “Top-down” methods rely on generating voids in monocrystalline silicon wafers using the chemical and/or physical removal of atoms from the silicon substrate to create highly directional porosity. “Bottom-up” approaches, on the other hand, depend on assembling silicon clusters together in a way that, while establishing a crystalline form, leaves voids behind so that a porous structure can be synthesised [55]. Most processes for obtaining pSi over the last 50 years have relied on electrochemical anodisation of monocrystalline silicon wafers in aqueous electrolytes comprised of ethanol and hydrofluoric acid (HF) [56,57]. In electrochemical etching, the solid silicon wafer functions as an anode, while a platinum (Pt) plate functions as a cathode when both are submerged in the HF-ethanol electrolyte (Figure 4). The intrinsic properties of the pSi obtained through this route, such as pore size and shape, pore layer thickness, and porosity, are mainly determined by the manufacturing conditions. These conditions include current density, wafer type, resistivity, HF concentration, chemical composition of the electrolytes, crystallographic orientation, temperature, time, electrolyte stirring, illumination intensity, and wavelength. While complete control over all of these process parameters is a major challenge for the fabrication of pSi, most of them are somehow related and can be kept constant, thereby achieving a satisfactory degree of reproducibility during the whole process [58]. 

### 1.5. Porous Silicon Surface Stabilisation

Following anodisation, the pSi surface becomes hydrogen-terminated (Si–H_x_) and displays a certain degree of environmental chemical reactivity. The bonds at the surface can be Si–H, Si–H_2,_ and Si–H_3_ hybrids and render the silicon surface prone to oxidation even in dry ambient conditions [57,59,60]. In addition to the native oxidation of pSi, the extent and rate of oxidation is dependent on the storage conditions, with the transition from hydrophobic hydrogen termination to the hydrophilic oxidised surface taking place over the course of months at room temperature. Complete native oxidation, however, occurs over a much longer time period [61,62]. 

**Figure 5:**
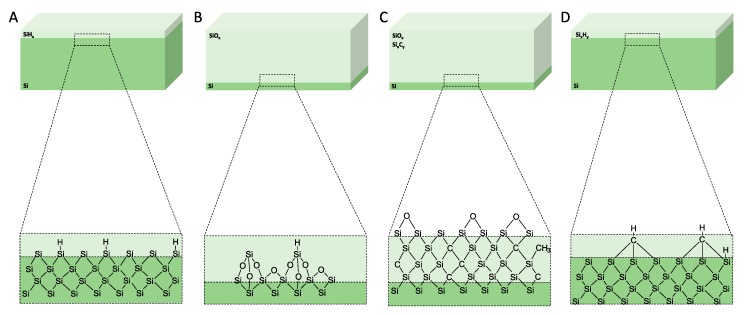
Schematics of the surface chemistry of the mesoporous materials after anodisation and after surface treatments. (**A**) As-anodised, (**B**) oxidised, (**C**) carbonised, and (**D**) hydrocarbonised. Reproduced with permission from [65].

The silicon hydride species present in the as-anodised surface of pSi can promote reactivity towards any compound potentially loaded within the pores of pSi [63,64], and thus, a stable nonreactive surface is essential to replace the unstable hydrogen-terminated surface of the freshly etched pSi. The conversion of the reactive groups at the surface into more stable oxidised, hydrosilylated, or (hydro) carbonised forms allows for further modification of the pSi surface (Figure 5), which can include radiotracers such as ^18^F [65]. Selective substitution of hydrogen on the surface of pSi with hydroxyls can also be conducted to leverage direct chemical linking of the pSi with organic molecules, therefore circumventing the intermediate oxide layer. This can be achieved either by: (1) selective oxidation of Si–H groups using diluted solutions of hydrogen peroxide in water, with the addition of 15% methanol or 10% acetone to render the solution capable of wetting the hydrogen-terminated surface of pSi and penetrating into the pore volume, or (2) by direct or indirect substitution of the surface-bound hydrogen with a hydroxyl via intermediate halogenation of the pSi surface by a radical mechanism and directly as a result of the nucleophilic substitution of the hydride ion (H–) with a hydroxyl anion from an alkaline solution [66].

#### 1.5.1. Thermal Oxidation

One of the most straightforward methods to oxidise pSi surfaces is through thermal oxidation, which gives rise to thermally oxidised pSi (TOpSi) [67,68]. The thermal oxidation of the pSi surface occurs at a threshold temperature of around 250 °C under ambient air conditions where the loss of hydrogen from the pSi surface is first detected and where the Si dangling bond sites generated are able to chemisorb O_2_ dissociatively, leading to the first stage of surface oxidation. This initial oxidation promotes further insertion of oxygen into the Si–Si back-bonds, giving rise to –O_y_SiH_x_ species. Concurrently, the oxidation process also leads to the formation of surface Si–OH species as a result of the oxygen insertion into the Si–H bonds [69]. The conversion of the native Si_y_SiH_x_ surface to O_y_SiH, O_y_SiOH, and SiOSi through thermal oxidation has been amply reported in the literature, especially due to its effect on pSi photoluminescence [69,70,71]. In addition to thermal oxidation, there are various other ways to obtain a pSi surface oxide. These include maintaining the pSi under an atmosphere of oxygen [69], a short exposure to ozone [72], and treatments with dimethyl sulfoxide [73] or hot concentrated solutions of hydrogen peroxide [74].

#### 1.5.2. Hydrosilylation

Hydrosilylation is another process of passivating the silicon surface and can summarily be described as a radical-induced reaction which can be initiated either by free radical initiators, ultraviolet light (UV), or thermal energy. The latter case produces a covalent linkage of alkyl chains to the hydrogen-terminated pSi surface, using unsaturated compounds such as terminal alkenes and alkynes as substrates. The first reports of a covalent linkage of densely packed, long alkyl chains directly to a silicon surface were published by Linford and Chidsey, where they employed pyrolysis of diacyl peroxides in the presence of hydrogen-terminated silicon. The authors used the thermal decomposition of diacyl peroxides to produce alkyl radicals, CH_3_(CH_2_)_n_^•^ via CH_3_(CH_2_)_n_COO^•^, which were then reacted with the silicon surface. They also concluded that a large number of the linkages formed using this process were C to Si direct bonds. However, it was also noted the presence of some carbonyl groups, which indicated that these monolayers were not comprised solely of alkyl chains. This was attributed to the presence of hydrolysable acyloxy bonds to the silicon surface (Si–O–C(O)–CH_2_–) for one-third of the chains, whereas the more robust alkyl bonds to the silicon surface (Si–CH2–) comprised the remaining fraction of the chains [75]. The same group later reported an optimised method for obtaining chains packed at approximately 90% the density of crystalline *n*-alkanes using pyrolysis of mixtures of either 1-alkenes or 1-alkynes and diacyl peroxides [76]. The presence of stable Si–O bonds at the surface decreases the number of reaction centres, since these bonds cannot be broken by UV light or by the thermal energies typically used in hydrosilylation reactions. By employing hydrogenated amorphous silicon (a—Si:H), a material known to be more resistive against oxide formation than crystalline silicon [77] for the fabrication of hydrosilylated silicon surfaces, the additional benefit of allowing the substrate to be treated under ambient conditions during surface functionalisation could be achieved [78]. Lewis acid-mediated hydrosilylation of alkynes and alkenes on nonoxidised hydride-terminated porous silicon has also been reported, where EtAlCl_2_ acts upon terminal, cis- and trans-disubstituted, trisubstituted, and tetrasubstituted alkenes and terminal and internal alkynes to promote the covalent attachment of organic functionalities to the silicon surface via Si–C bonds, resulting in alkenyl- or alkyl-terminated surfaces [79,80,81]. A hydrosilylation approach towards the formation of Si–C bonds on silicon surfaces, which yielded surface-bound vinyl and alkyl groups, respectively, has also been described. The authors employed a Pt-catalysed reaction between 3,4,-dichlorobutene and hydrogen-terminated silicon surfaces, where after an initial step involving adsorption of Pt on the hydrogen-terminated silicon, an oxidative addition of the SiH to the coordination sphere of the Pt(0) complex to form (dichlorobutene)_3_Pt^2+^ (H)(Si) occurs. Subsequently, hydride addition to dichlorobutene takes place during the migratory insertion step, which is followed by the reductive elimination of the alkylsilane [82]. The pSi surface can also be hydrosilylated by reductive electrolysis of organohalides, where the pSi is immersed in a solution containing an organohalide (RX, X=I, or Br) and then passing a cathodic current through the solution. The hydrosilylation occurs either by direct reaction between the Si radical and the alkyl radical or by reduction of the Si radical to an anion, followed by a nucleophilic attack of the organohalide [83]. This hydrosilylation route has been reported to functionalise 20%–80% of the Si–H bonds on the pSi surface, which still leaves the remaining Si–H bonds vulnerable to attack and oxidation. However, by following the organohalide functionalisation with a CH_3_I methylation of the remaining Si–H bonds, it is possible to achieve a larger Si–C surface coverage [84].

#### 1.5.3. Thermal Carbonisation 

Thermal carbonisation is another process employed for stabilising the silicon surface, which also involves chemical derivatisation of the pSi surface with organic compounds and formation of Si–C bonds [85]. It was first described in the late 1990s as an attempt to stabilise the photoluminescence of pSi and, although the treatment does indeed produce a stable nonstoichiometric silicon carbide layer on the pSi surface, it also completely quenches its initial photoluminescence [86,87]. Early efforts relied on reactions involving heating of pSi single-crystals, which in turn proved potentially damaging to the fragile nanoscale structure of the substrate [88]. Several research groups have subsequently investigated the formation of Si–C bonds on Si at room temperature using one- or two-step methods [79,89,90,91]. One-step derivatisation reactions in Si usually entail photo- or electrochemical methods to attach one or two carbon fragments to the surface (CH_3_–Si and RCO_2_–Si, R=H, and CH_3_), whereas in the two-step method developed by Lewis and collaborators, the H-terminated Si surface was first chlorinated radically by PCl_5_ and subsequently quenched with a Grignard reagent [91].

The pSi surface produced via thermal carbonisation is remarkably stable to boiling alkali solutions, which indicates a sufficient degree of coverage to fully protect the exposed surface, whereas the Si–H-terminated pSi dissolves quickly under these conditions [89]. Stability studies of differently stabilised pSi samples have shown that the thermal carbonisation of pSi (TCpSi) is an even more efficient stabilising method than thermal oxidation [92]. Functionalisation of TCpSi by radical coupling of sebacic acid has been also reported, as well as their capability to further modify the pSi surface using standard bioconjugate chemistry methods. The surface was stable and comparable to a non-functional thermal oxide but superior to the widely used carboxy-terminated surface prepared by the thermal hydrosilylation route [93]. 

#### 1.5.4. Thermal Hydrocarbonisation 

Thermal decomposition of acetylene is another method that can be used to generate a hydrocarbon-terminated surface [94]. This surface treatment allows functionalisation to be carried out at a lower temperature, as there is a threshold temperature which changes the thermal functionalisation of pSi into carbonisation. This temperature, nonetheless, allows for acetylene to be continuously circulated without the problem of graphitisation. Below 700 °C, the hydrogen atoms remain on the pSi surface, rendering it hydrophobic (Si–C–H bonds, thermally hydrocarbonised pSi, and THCpSi). Temperatures above 700 °C, on the other hand, allow the dissociation of hydrogen from the surface, rendering the surface more hydrophilic than the material produced at lower temperatures. One of the advantages of using small gaseous molecules of acetylene includes the faster and improved diffusion into the pores, which further improves the efficiency of surface coverage. The hydrocarbon-terminated surface Si–C–H can be present in several configurations, with three silicon atoms bonded to a C atom presenting as the most common. This surface treatment has several advantages over the carbonisation treatment, including: the pSi surface remaining hydrophobic, the treated layer is thinner, and the gas adsorption properties are different from those found in the carbonised pSi. This treatment also enables further functionalisation of the pSi for cell-targeting and antifouling purposes [95,96]. 

### 1.6. Metalothermic Reduction

Other than etching, chemical conversion of silica is nowadays one of the most prevalent routes for obtaining pSi [97]. Nanostructured silica can be reduced to pSi with the assistance of reducing agents such as magnesium and aluminium at moderate temperatures (400–800 °C). The milder reduction temperatures offer a desirable alternative for producing silicon when compared with the industrial blast furnace carbothermic reduction method (over 1400 °C) [98]. Magnesiothermic reduction of silica was first described in the early 1980s in India when researchers tried to obtain solar-grade silicon from rice husks [99,100]. The process of magnesiothermic reduction can be briefly detailed as follows: starting at a temperature of 400-600 °C in an inert atmosphere or vacuum, magnesium gas reacts with silicon dioxide to yield silicon and magnesium oxide according to the chemical reaction depicted in Equation (1):

(1) $$SiO_{2\;}\left(s\right)+2\;Mg\;\left(g\right)\to Si\left(s\right)+2\;MgO\;\left(s\right)$$

The magnesia (MgO) can be easily removed afterwards with HCl, leaving behind a silicon replica with higher surface area than the starting template. The exothermic nature of the reduction reaction allows for utilisation of the heat produced, aiding the process further and lowering the cost of the reaction [101]. 

It is, however, noteworthy to point out that a side reaction can reduce the yield of silicon through the formation of magnesium silicide (Mg_2_Si), as depicted in Equation (2). The Mg_2_Si is obtained when the gaseous magnesium reacts with silica on the surface, and, consequently, the formed Si product prevents access of magnesium to silica in the interior, causing a mismatch of the stoichiometric ratio of magnesium and silica, thus resulting in an undesired side reaction that produces magnesium silicide [102]. The formation of Mg_2_Si reduces the yield of Si formed and impacts on the morphology of the final product upon removal, which occurs concurrently with the MgO removal with the HCl wash [103].

(2) $$Si\;\left(s\right)+2Mg\;\left(g\right)\to Mg_{2}Si\left(s\right)$$

The greatest advantages of the magnesiothermic reduction of silica is the inexpensive silica feedstocks that can be employed (sand, rice husk ash, etc.), as well as the lower required amounts of HF or organic solvents throughout the process. Another reducing agent that has been given increasing attention is aluminium. Aluminium is an inexpensive metal which has long been known to react rapidly with silica to yield silicon, according to Equation (3).

(3) $$3SiO_{2}\left(s\right)+4Al\left(l\right)\to 3Si\left(s\right)+2Al_{2}O_{3}\left(s\right)$$

The advantages of using aluminium, besides its lower cost compared with magnesium, are the avoidance of minor silicide by-products (such as the Mg_2_Si described earlier) and the alumina passivation of the silicon structure [104]. 

The magnesiothermic reduction of silica nanoparticles has been reported to increase the surface area from 8.2 m^2^g^−1^ to 386 m^2^g^−1^ after a 12-h reaction time at 500 °C. Pore volume also increased from 0.07 cm^3^g^−1^ to 1.7 cm^3^g^−1^. The surface area of silicon particles produced by aluminothermic reduction was a magnitude smaller than the magnesium-reduced product, with the greatest surface area reported as 37 m^2^g^−1^ after 24 h at 650 °C. Pore volume was determined to be 0.51 cm^3^g^−1^ after 12 h at 650 °C. Transmission electron microscopy (TEM) images of the porous substrate showed that a aluminothermic reduction produces a less porous substrate than magnesiothermic reduction, with the porous silicon composed of layers [105].

### 1.7. Drug Loading and Release from pSi

As mentioned previously, there are many advantages of confining pharmaceutical compounds into mesoporous materials. To leverage this in the case of pSi, drugs need to be loaded into a mesoporous matrix in a reproducible fashion and in high yield. The most common methods for drug loading in the context of pSi materials are solvent loading, where the mesoporous matrix is immersed into a saturated solution of the drug [106,107,108], melt intrusion, where the mesoporous matrix is put into contact with the molten drug substance which facilitates complete pore-filling through capillary action [109], or even by supercritical drying of ultrahigh porosity (90%) pSi [110]. When assessing the degree of drug loading, it is critical to distinguish the fraction of drug within the pores from the drug on the external surface of the material. Several methods have been employed to quantify drug loading, such as calorimetry [30,111], high-performance liquid chromatography (HPLC) [107,108], atomic force microscopy (AFM) together with Time-of-Flight Secondary Ion Mass Spectroscopy (ToF-SIMS) [112], gas sorption [64], and XRPD. While there is not a single method that could discern between loaded and surface-bound drugs in pSi, a suite of thermoanalytical and spectroscopic techniques has been proved to provide valuable information on the physical state of the drug molecules.

**Figure 6:**
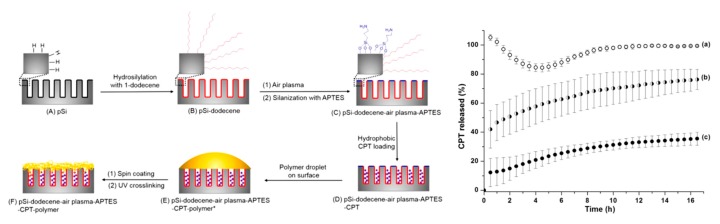
Schematic diagram showing the differential functionalisation of interior and exterior surfaces of pSi films to allow improved camptothecin (CPT) drug loading and release and CPT release profiles from (**a**) pSi-ozone-APTES, (**b**) pSi-dodecene-air plasma-APTES, and (**c**) pSi-dodecene-air plasma-APTES-2 wt% poly(HPAm-co-BPAm). Reproduced with permission from [114].

As previously mentioned, surface modification of pSi makes the substrate adaptable for the confinement of many compounds, both hydrophilic and hydrophobic. Loading capacity can increase if the pore surface is tailored to suit the functional groups of the loaded molecule. It has been reported that hydrophobic drugs load better into hydrophobic pores, but the drugs could prove difficult to wet in physiological conditions [113]. To try and address this, a three-step pSi functionalisation method was employed. Firstly, the pSi films were hydrosilylated with 1-dodecene, followed by etching of the surface and a further hydrosilylation stage using (3-aminopropyl)triethoxysilane (APTES). This process provided the outer surface with hydrophilic functional groups and the inner pore surfaces with hydrophobic groups, which suited the hydrophobic drug camptothecin. An additional layer of polymer was attached to the loaded pSi surface to further control the release of the drug. Modification of the pSi doubled the drug-loading concentration, and the external polymer coat improved the drug release profile with a slower, steadier release than the control (Figure 6) [114]. 

Nanoparticles of pSi have also been employed for drug delivery using polymer coatings, forming porous silicon-polymer composites. Krepker and Segal detailed the many designs of pSi-polymer composites and their fabrication, endowing the surface of the pSi with a wide range of surface chemistries for a variety of applications [115]. Porous silicon polymer nanocomposites have been used for the delivery of peptide nucleic acids (PNA) due to their large loading capacities [116]. However, pSi alone does not allow the release of the nucleic acid from the endo-lysomal vesicles into the cytoplasm and, therefore, requires additional modifications. Oxidised pSi nanoparticles were loaded with PNA before the application of a polymer coating, poly((ethylene glycol)–block–(2-(dimethylamino)ethyl methacrylate–co–butyl methacrylate)) (PEGDB). The PEGDB allowed for the systemic delivery of negatively charged nucleic acids, facilitating endosomal release of the nucleic acid [117]. The modified surface of the pSi nanoparticles led to increased stability and reduction in particle aggregation in comparison to the uncoated pSi nanoparticles. It was stated that the increased stability witnessed was due to the polymer-blocking surface adsorption of proteins and ions, therefore preventing aggregation [118].

Salonen and collaborators looked at ibuprofen confined within thermally carbonised pSi to assess the loaded drug in comparison to drug crystallised on the surface by DSC [30]. Ibuprofen loaded onto nonporous silicon showed a melting peak (endotherm) at 74 °C, close to that of the bulk drug (76 °C). Ibuprofen loaded in pSi and washed in a solution of water and ethanol displayed two endotherms in the thermograph: the suppressed melting point of confined crystalline material and a small amount of surface crystals with a melting peak close to that of bulk ibuprofen. Washing the loaded pSi in pure ethanol showed no surface crystallisation. Depression of the melting point is often observed for confined crystalline compounds due to the reduced size of the crystals [119]. The confinement and increased drug dissolution has been further confirmed with studies which included the poorly water-soluble drugs indomethacin and griseofulvin, with the amorphous state of the confined compounds enhancing the drug dissolution or drug permeation rate [19,107,120,121,122]. In these studies, the loading degree of both drugs ranged between 6 to 29 wt% for indomethacin and 6 to 17 wt% for griseofulvin, depending on surface chemistry and pore size of the pSi substrate and highlighted the potential impact of these physicochemical parameters in further pharmaceutical processing. The loading of poorly water-soluble antiviral compounds, such as saliphenylhalamide, has also been conducted, in which a 2.88% drug loading was reported [108]. The authors attributed the low loading values to the drug’s partition coefficient (a measure of the lipophilicity of a compound) and its molecular radius. 

The application of pSi to deliver the anti-cancer drug doxorubicin to transferrin receptor-overexpressing tumour cells across a blood-brain barrier (BBB) model has also been reported [123]. The pSi particles were first functionalised using undecylenic acid to produce particles with carboxyl terminations and further conjugated to transferrin to target cell surface markers. The hypothesis of this study was that the transferrin-coupled pSi particles would be able to cross the BBB and, furthermore, bind to transferrin receptors found on the surface of tumour cells to deliver the drug. Unlike many other studies involving the use of pSi as a drug delivery system, the focus of this investigation was not to increase the solubility of a poorly aqueous soluble drug but to achieve targeted drug delivery using functionalised pSi particles. The functionalisation of the silicon surface increased the particles’ colloidal stability, as demonstrated by a decrease in hydrodynamic radius measured by dynamic light scattering. Loading was determined to be 87.90 ± 2.16 μg/mg pSi particles, with drug release dependent on the pH of the environment. The release of the drug was studied to simulate endolysosomal and physiological conditions (pH 5 and 7.4, respectively). Stable hydrogen bonds were formed between the pSi surface and doxorubicin in a neutral pH, limiting the drugs release. Hydrogen bonds weakened in an acidic environment, leading to a greater drug release, with up to 90% in 12 h. Furthermore, brain tumour tissue is known to have a lower pH than normal tissue; therefore, a pH-dependent rate would be beneficial for improved drug release at the target site [123].

The successful confinement of crystalline and amorphous small drug molecules has led to research into the confinement or adsorption of proteins to mesoporous substrates, an area of increasing promise to the pharmaceutical industry [33,35,107,124].

The use of pSi as an aid in the crystallisation of proteins has also been investigated due to the limited success of protein nucleation on other modified surfaces. The pSi was used as a substrate on the basis that the pores would enable the confinement of protein molecules, which would lead to nucleation and crystal growth. The average pore size of the material was between 5 and 10 nm with a solution-loading method employed. The pSi induced the nucleation of five out of the six proteins (lysozyme, trypsin, catalase, thaumatin, and phycobiliprotein) with a hydrodynamic radii ranging from 2 to 5 nm, with crystals either growing on the silicon fragments or growing within the oil droplet which suspended the silicon and protein solution, decreasing in size the further away they were from the silicon fragment. This could have been due to nuclei diffusing away from the nucleation site or nucleation taking place on smaller fragments of pSi. An explanation as to why concanavalin A, one of the proteins, did not crystallise in the presence of the pSi substrate was not discussed. Further to this, the influence of smaller pore sizes on crystallisation was investigated using other porous materials, which did not prove successful. This has supported the hypothesis of the dependency of pore size on the crystallisation of proteins. The crystallisation of proteins usually depends on the pH of the system; however, using pSi as a nucleation surface removed this dependency with confinement of the molecules, leading to localised supersaturation—hence, enabling crystallisation [33]. 

**Figure 7:**
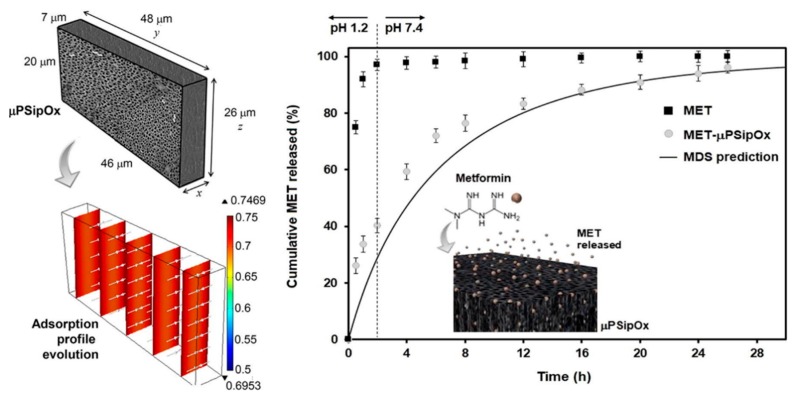
Representative geometry used for modelling the loading kinetics during the adsorption of metformin (MET) onto porous silicon microparticles (μpSip), dimensionless concentration profiles inside the μpSipOx during the adsorption of MET, and in vitro release profiles of pure MET and MET-μpSipOx (oxidised porous silicon microparticles). Reproduced with permission from [126].

The loading of metformin into pSi for extended drug release has also been investigated. Metformin is routinely administered in high and frequent doses, which can cause side effects within the gastrointestinal tract. Attempts to increase the bioavailability of the drugs have been employed, including research into the encapsulation of metformin into chitosan-poly(lactide-co-glycolide) nanoparticles only producing low encapsulation efficiency values [125]. The pSi offered the sustained release needed due to a combination of morphological features and surface chemistry. The aim of the work was to investigate the bonding between drug and carrier, an area which many other studies have failed to address. The pSi was fabricated from electrochemical etching of silicon wafers with surface passivation via thermal oxidation to promote the adsorption of the drug. The largest dimension of the pore was determined to be 8 nm ± 2 nm. Due to the irregular shape of the pores, the average pore diameter was not conclusive. Albeit, the pores were large enough to facilitate the free movement of the drug into the pores and through the porous network. Interestingly, the authors analysed the distribution of charge within the drug-loaded pSi and found that metformin was protonated in media with a pH less than 2.8, monoprotonated between pH 2.8 and 11.5, and neutral above pH 11.5. The pH of the media also influenced the surface chemistry of the oxidised pSi, which therefore influenced its drug-loading capacity. Particles displayed a positive surface charge at a pH of 2.2 or lower and negative charge above that value. A zeta potential of less than 30 mV indicates particle instability, which was determined to be at a pH less than 7. This provided an optimum pH of 7, with the negatively charged surface of the pSi (Si-O^−^) favouring the loading of the positively charged metformin molecules, with electrostatic forces enabling the interaction between the drug molecules and the carriers’ surface (Figure 7). Overall, this study found thermally oxidised pSi to be advantageous as a carrier of metformin, with prolonged release over a period of 26 h [126]. 

The use of pSi as a carrier for fatty acid α-linolenic acid has also been reported [127]. The aim of the study was to load compounds of nutritional benefit into a drug carrier in order to stimulate the secretion of gut hormone glucagon-like peptide 1 (GLP-1). Nutrients such as fatty acids have been known to aid the regulation of appetite and blood glucose levels, which is beneficial in the treatment of obesity [128]. The pSi particles were first thermally hydrocarbonised to aid the loading of the hydrophobic fatty acid and delay its release. Two drug-loading ratios were used within the study: 3% and 9.2%; however, 49.4% ± 6.29% and 25.7% ± 1.53% of the confined α-linolenic acid remained unreleased, respectively. It was stated that the empty pSi did not stimulate to expression of GLP-1 from the STC-1 cell line, but the fatty acid-loaded substrate increased GLP-1 secretion by 1.5% compared to buffer alone. The detectable amount of α-linolenic acid was reduced with increased incubation time and acidity of the buffer, which may have been due to the accelerated oxidation of fatty acid in an acidic environment [129]. However, the time in which fatty acids remain within the acidic environment of the human stomach depends on many factors, including the type of food and how often it is consumed. 

Microparticles of pSi loaded with GLP-1 were also reported to lower the blood glucose levels after a single subcutaneous injection in mice, but none of the microparticles were able to prolong the glucose-lowering effect, as compared to the GLP-1 solution [130]. In this study, the authors loaded and released the GLP-1 peptide from negatively charged TOpSi and TCpSi microparticles and from positively charged amine modified microparticles, designated as TOpSi-NH2-D (isolelectric point 8.8) and TCpSi-NH2-D (isoelectric point 8.8), respectively. They found that the adsorption of GLP-1 onto the pSi microparticles could be increased 3–4-fold by changing the pSi surface charge from negative to positive, indicating that the positive surface charge of pSi promoted an electrostatic interaction between the negatively charged peptide. The adsorption and desorption kinetics of the GLP-1 peptide were also subject to study, where the authors concluded that, albeit electrostatic attraction between the peptide and the pSi surface is relevant, at low concentrations, the hydrophobic interaction seems to have a higher impact, even though the effect is less important for most hydrophilic nanoparticles [131].

Molecular modelling has been used to identify the intermolecular interactions that characterise physicochemical factors, influencing the release of a drug from a dosage form [132]. Polkovnikova and collaborators aimed to investigate the activation energy required for drug desorption from silicon and silicon–dioxide surfaces. Desorption and dissolution of the drug from the surface was observed when the van der Waals interaction energy reached zero. The activation energy was defined as the difference between the average energy of the desorbed and adsorbed state in water. The change in electronic energy was relative to the initial state, with the drug either in its ionised or unionised state when adsorbed to the surface.

The unionised drug molecules were positioned flat on the surface of the silicon. Desorption of the drug from the surface was said to occur in two stages: first, the separation of the polar part of the molecule, followed by the separation of the hydrophobic phenyl radical, which was stated to have a greater affinity for the silicon. Desorption energy profiles showed evidence of these two stages with a two-stage energy increase. The ionised drug desorbed quicker than the unionised drug, also occurring in two stages; firstly, the hydrophobic phenyl group attached to the SiH and SiOH groups of the silicon surface and, then, the subsequent removal of the hydrophilic section of the drug. Bonding between the drug and adsorbent surface was also investigated, with the hydrogen bond between the surface OH group of the SiOH and the nitrogen atom of the drug breaking during desorption for both ionised and unionised surfaces.

Estimations of the activation energies for the dissolution of the drug from the surface of the adsorbent into water were calculated by using the difference between the electronic energies of the system dissolved in water and the initial adsorbed state. The energy required for desorption of the drug from the ionised SiO_2_ surface was overall the greatest, with desorption of the drug cation lower than that of the drug in its unionised state (110.66 and 347.30 kJ/mol, respectively). Estimations of the activation energy of drug desorption in aqueous solutions of different pH values were also investigated. Drug desorption from the SiO_2_ surface at pH 6.8 and 7 showed the greatest statistical significance, which is significant if dissolution was to occur in the large intestine. 

The ability to load and release drug molecules trapped within its pores has led to discussions over the possibility of employing pSi as a nanoscale delivery system but also raised concerns about their toxicological profile in a biological setting [133]. Ever since pSi was first reported as a bioactive material [48], many studies have been devoted to study its biocompatibility and biomedical applications [134,135,136,137,138,139,140]. It has been reported that pSi biodegradation can be controlled by the overall porosity, pore size, shape, surface, and bulk properties [141,142], which in turn can be modulated by controlling the material fabrication parameters of the matrix [143,144]. For instance, pSi with a porosity >70% dissolves in all the simulated body fluids (except gastric fluids), whereas pSi with a porosity <70% is bioactive and slowly biodegradable [52]. The fact that pSi degrades mainly into monomeric silicic acid, Si(OH)_4_, the most natural form of Si in the environment and very important in human physiology in protecting against the poisonous effects of aluminium [145], is an important feature that contributes even further to the pSi apparent biocompatibility. It has been reported that the average intake of Si is approximately 25–40 mg/day [146,147] and that Si is an essential nutrient for the human body. Blood concentrations of Si(OH)4 have been found to be slightly above the typical values of 1 mg/L [145], and, most importantly, silicic acid does not accumulate within the human body and has been shown to be absorbed readily by the gastrointestinal tract of humans and rapidly excreted via the urinary pathway [148].

## 2. Summary

Mesoporous silicon has shown many advantages as a drug carrier due to its large loading capacity, tuneable pore size, controlled drug release, and the ability to adapt the surface to suit the drug to be loaded. Porous materials can influence the polymorphic form of the confined crystalline state or stabilise the disordered amorphous state confined within them, which shows promise as a way of improving the dissolution rate of poorly water-soluble drugs. Protein crystallisation has also successfully utilised porous materials for heterogeneous nucleation, using porous silicon as a templating surface.

From this review, we have been able to identify the issues faced by many researchers in their investigations into the confinement of drug compounds in mesoporous materials; for example, crystallisation on the surface of the porous substrate limiting the overall drug dissolution. A clear understanding is needed of how external factors such as temperature and pH influence crystallisation, as well as the pore structure and surface chemistry of the substrate, before translation to the pharmaceutical industry. The nucleation of the crystalline state in porous silicon needs to be evaluated, including the influence of pore diameter and pore volume, as well as the interactions between drug molecules and the surface of the substrate. Neutron and synchrotron X-ray diffraction, as well as solid-state NMR, are emerging as advanced characterisation techniques that are now beginning to be utilised to tackle this, along with the conventional methods such as DSC and XRPD. 

These fundamental issues need to be addressed continuously in order for research to turn to the scale-up of these processes and make drug confinement in mesoporous materials feasible for pharmaceutical development.
